# Effectiveness of Composite Ayurveda Regimen in a Black Box Design for the Management of Rheumatoid Arthritis: Protocol of a Single Arm, Community-Based Study

**DOI:** 10.2196/57918

**Published:** 2025-02-18

**Authors:** Deepa Makhija, Sunita Mata, Abha Sharma, Kalpana Kachare, Aparna Manathottathil, Seema Jain, Sophia Jameela, Bhogavalli Chandrasekhara Rao, Rakesh Rana, Arunabh Tripathi, Kiran Rana, Vandana Joshi, Anukampa Singh, Narayanam Srikanth, Rabinarayan Acharya

**Affiliations:** 1 Central Council for Research in Ayurvedic Sciences New Delhi India; 2 Central Ayurveda Research Institute Delhi India

**Keywords:** rheumatoid arthritis, Amavata, Ayush-SG (coded drug), Rasnasaptak Kashaya

## Abstract

**Background:**

Rheumatoid arthritis (RA) is an autoimmune disease that affects joints and can have extra-articular manifestations. RA usually tends to be progressive and leads to substantial health care burdens, both in terms of disability and economic costs. Despite the various treatment modalities available, there is still an urgent need for safe and effective medicine based on the pattern of disease presentation. The increasing interest in complementary and alternative medicine has created a demand for extensive research in this area.

**Objective:**

This clinical study is designed to evaluate the effectiveness and tolerability of a composite Ayurveda regimen in RA.

**Methods:**

The study is a single-arm (pre-post design), community-based interventional study with a black box design being conducted at 6 study centers. A total of 240 participants aged between 18 and 65 years, diagnosed with RA as per the 2010 American College of Rheumatology and the European League Against Rheumatism criteria are recruited as per the selection criteria. All the participants received Ayush-SG and *Rasnasaptak Kashaya* for 84 days along with customized treatment as per the disease presentation and associated complaints. The outcome measures include the change in disease activity score (DAS)-28 with erythrocyte sedimentation rate, disease-specific biochemical and inflammatory markers, Disability Index score, change in the participant’s assessment of pain and frequency of use of conventional analgesics or nonsteroidal anti-inflammatory drugs from baseline. The tolerability of interventions is assessed through the occurrence of adverse events. Categorical variables will be analyzed with McNemar chi-square test, and continuous variables will be assessed using the paired *t* test or Wilcoxon test for pre-post assessment. The level of significance will be 5%.

**Results:**

The recruitment of participants was initiated in December 2023. The participant recruitment was completed in March 2024 and out of 240 participants enrolled, 222 (92.5%) completed the study up to the last follow-up. Data verification, compilation, and analysis are under process. After data analysis, the study’s findings will be published in a peer-reviewed journal.

**Conclusions:**

This interventional study that incorporates the black box approach may provide a strong framework for managing RA. This design is a more reliable method for evaluating the effectiveness and tolerability of the composite Ayurveda regimen in RA.

**Trial Registration:**

Clinical Trial Registry-India CTRI/2023/06/054203; https://tinyurl.com/4prvwr6z

**International Registered Report Identifier (IRRID):**

DERR1-10.2196/57918

## Introduction

### Background

Rheumatoid arthritis (RA) is a chronic inflammatory autoimmune disease that can damage joints and affect extra-articular organs. In 2019, 18 million people worldwide were living with RA. Women accounted for 70% of cases, and over half were older than 55 years [1]. The articular manifestation of RA includes symptoms such as musculoskeletal pain, swelling, and stiffness of joints. It is usually symmetric and initially manifests in small joints and progresses to larger joints. Over time, joint inflammation can lead to joint destruction, including loss of cartilage and bone erosion [2]. The symptoms of RA vary in patients; some patients have mild self-limited disease, while many experience joint destruction, severe physical disability, and multiple comorbidities. RA tends to be progressive in nature, involving a worsening of symptoms over time, and often begins for many people during the early or middle years of life, thus causing a heavy burden on society in terms of disability, health, and economic costs. The available evidence for pain management in RA involves the use of disease-modifying antirheumatic drugs and medications such as nonsteroidal anti-inflammatory drugs (NSAIDs) which play an important role in its management but may suppress the immune system and, lead to an increased risk of infections as well other side effects as gastrointestinal disturbances [3,4]. People from all over the world are also developing an interest in traditional herbal practices. It is reported that 60% to 90% of persons with arthritic conditions use complementary and alternative medicines [5-7]. In a study conducted in the United States, it was reported that around 60% of persons with arthritic conditions including RA use complementary and alternative medicines. Of these, 28% reported a history of herbal therapies, and 22% used diet supplements [8].

RA resembles the condition “*Amavata”* (rheumatism due to Ama) described in Ayurveda*,* where both the *Ama* (undigested or intermediate product of digestion or metabolism that act as a toxin in the body) and *Vata Dosha* (*Dosha* responsible for movement and cognition) become vitiated and located in the *Sandhi* (joints) leading to pain and inflammation in the joints. The causative factors of Amavata are described as *Viruddha Ahaara* (antagonistic food), *Viruddha Vihaara* (antagonistic lifestyle), *Mandagni* (subdued digestive power), a sedentary lifestyle, and exercising immediately after meals. Long-term adherence to these etiological factors causes vitiation of *Vata* along with impaired digestion and metabolism in the body leading to the formation of *Ama*. The gradual accumulation of *Ama* obstructs different *Srotas* (structural or functional channels) in the body and further vitiation of *Tridoshas* (3 regulatory functional factors of the body). The Ama and Vata along with other vitiated Doshas lead to the impairment of *Dhatus* (major structural components of the body) mainly joints causing the *Amavata* [9].

The Ayurvedic classics describe various treatment modalities and therapeutic formulations based on the condition and clinical presentation of the disease. The disease pattern of *Amavata* in a person depends on the extent of *Ama* association, *Dosha* vitiation, and the chronicity of the condition. In cases of *Amavata* with significant *Ama* association, there may be obstructions in the *Srotas*, leading to symptoms like severe pain, swelling, elevated temperature, and redness in the joints. The treatment for this scenario should prioritize digesting *Ama* and clearing the obstructions in the *Srotas* along with alleviation of vitiated *Dosha*. If there is only mild *Ama* association and less *Dosha* imbalance, symptoms may be limited to joint pain, stiffness, and minimal swelling, indicating medications that digest *Ama* and alleviate *Doshas* predominantly *Vata*. In chronic cases, patients may experience symptoms like mild aching stiffness, fatigue, or anemia. The choice of medicines varies according to each pattern of presentation and must be determined by the practitioner following a thorough assessment of the patient’s condition. In a standard randomized controlled trial (RCT), it is not feasible to design a customized regimen that tailors medicine selection to specific conditions. In such instances, a specialized trial design should be considered to facilitate the prescription of medicines tailored to the clinical manifestations and disease stage of each individual. To address this issue, it is essential to consider modifying study designs, including exploring alternative approaches such as “black-box” designs [10].

The treatment plan for *Amavata* is focused on the digestion of *Ama* and the alleviation of *Vata*. For digesting the *Ama* and removing obstruction from the *Srotas*, drugs having *Katu* and *Tikta* Rasa (pungent and bitter tastes), *Pachana* (enhancing digestion), and *Dipana* (enhancing metabolic fire) properties have to be administered [11]. Ayush-SG, *Rasnasaptak Kashaya*, and *Guduci* (*Tinospora cordifolia*) have the above-said properties. Ayush-SG is a coded drug developed by the Central Council for Research in Ayurvedic Sciences (CCRAS) for the management of Amavata [12]. *Rasnasaptak Kashaya* is described in the context of *Amavata* treatment in Ayurveda classics [13]. *Brihat Saindhawadi Taila* and *Dashang Lepa* are used for local application to pacify pain and inflammation of the joints [14-16]. Considering the major patterns of disease presentations, a black box design has been planned for this study and the treatment has been tailored according to the accompanying symptoms.

### Objectives

The primary objective of the study is to evaluate the effectiveness of a composite Ayurveda regimen on the disease activity score (DAS) in RA.

The secondary objective is to evaluate the effectiveness of the composite Ayurveda regimen on the disease-specific biochemical and inflammatory markers and assess the tolerability of Ayurveda regimen in RA.

## Methods

### Study Design

This is a single-arm (pre-post design), community-based interventional study with a black-box research design.

### Study Setting

The study is conducted through 6 research institutes under CCRAS at New Delhi, Patiala, Guwahati, Gwalior, Vijayawada, and Chennai in identified areas predominantly dwelled by the scheduled caste population near the institute.

### Inclusion Criteria

Participants aged between 18 and 65 years, diagnosed with RA as per the 2010 American College of Rheumatology and the European League Against Rheumatism criteria [17], and willing to give written informed consent for participation are enrolled in the study.

### Exclusion Criteria

Participants presenting with complications of RA, for example, deformity of joints/bones, pleura-pericardial disease, participants with extra-articular manifestations of RA and gastrointestinal symptoms, those with joint prosthesis or unable to walk without support or confined to a wheelchair, participants with Hemoglobin <8 g/dL, diagnosed with other arthritis like osteoarthritis, gouty arthritis, tuberculous arthritis, psoriatic arthritis, spondyloarthropathy, active fibromyalgia, juvenile chronic arthritis, ulcerative colitis, or other systemic inflammatory conditions and autoimmune diseases, having blood pressure ≥ 160/100 mm Hg, or hemoglobin A_1c_ (HbA_1c_)>8%, on medication with corticosteroids, antidepressants, anticholinergics, etc or Ayush interventions/folk medicine or any other drugs that may influence the outcome of the study are excluded from the study. Participants with diagnosed concurrent neurological, pulmonary, or endocrine disorder, or unstable cardiovascular disease, with concurrent serious hepatic disorder (defined as aspartate aminotransferase or alanine aminotransferase, total bilirubin, alkaline phosphatase > 2 times upper normal limit) or renal disorders (defined as S Creatinine > upper normal limit); participants with alcohol use disorder (CAGE score >2) or any other substance abuse; and pregnant or lactating woman are not enrolled in the study.

### Study Procedure

The participants have been screened from the out-patient departments, camps, or door-to-door visits in the identified areas. Before the initiation of the study, the head of the village or local administrative authority and the residents of the area were informed about the study in detail, in their regional language. Participants diagnosed with RA have been enrolled based on the defined inclusion and exclusion criteria.

**Figure 1:**
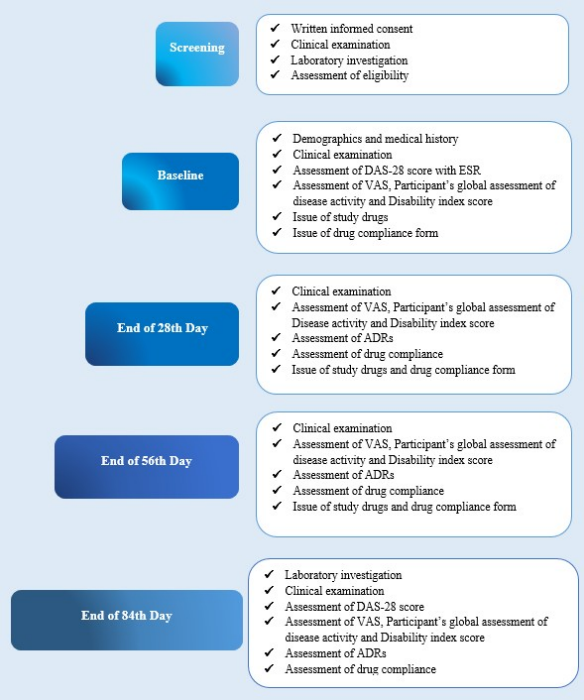
Flow diagram depicting the study schedule for the assessment of the effectiveness of composite Ayurveda regimen for the management of rheumatoid arthritis. ADR: Adverse Drug Reaction; DAS: disease activity score; ESR: erythrocyte sedimentation rate; VAS: visual analog scale.

Laboratory investigations such as complete blood count with an erythrocyte sedimentation rate (ESR), liver function test, renal function test, and C-reactive protein, RA factor (quantitative), serum immunoglobulin G, and serum immunoglobulin M have been conducted at baseline and the end of the treatment (84th day). HbA_1c_ levels are determined at baseline only (Figure 1)**.**

### Intervention

The treatment regime was customized based on the major patterns of disease presentation in each participant. All of them have been given oral medication Ayush-SG one gram in tablet form twice a day with lukewarm water after meals and *Rasnasaptak Kashaya* 20 mL twice a day (80 mL water added in 10 g of course powder and boiled until remains 20 mL) before meals for 12 weeks. Along with this, *Brihat Saindhavadi Taila* has been given for local application in cases with only articular manifestations (joint pain, stiffness, and minimal swelling). Participants with severe pain and swelling (boggy swelling or affusion elicited by fluctuation test), elevated body or joint temperature, and redness of joint, have been administered *Guduchi Ghana vati* (*Sanshamani Vati*) orally in the dose of 500 mg twice a day with lukewarm water after meals and *Dashanga Lepa* for local application. *Punarnavadi Mandura* has been given orally in a dose of 500 mg twice a day with lukewarm water after meals in participants with mild aching, stiffness, fatigue, and anemia.

Incidental medication has been given to the participants in case of any associated complaints. For constipation, *Triphala Churna* has been given in a dose of 5 g orally with lukewarm water at night before sleep. For *Mandagni*, *Vaishwanar Churna* has been administered in a 2.5 g dose orally with lukewarm water twice a day just after food. For the participants having insomnia/anxiety, *Ashwagandha Churna*, 3 g has been given orally twice a day. The duration of incidental medication is decided by the investigator as per the requirement.

The medicines were purchased from Ayurveda manufacturing pharmacies that are certified with good manufacturing practices. Ayush-SG was procured from the Central Ayurveda Research Institute’s Ayurveda Pharmacy, Jhansi, Uttar Pradesh, India. All other medicines administered in the study were procured from the Indian Medicines Pharmaceutical Corporation Limited (Mohan, Almora, Uttarakhand, India).

### Withdrawal Criteria

The participant has been withdrawn from the study if they have not been willing to continue, if there has been a worsening of RA symptoms, or if they have developed any other illness mentioned in the exclusion criteria. The decision to withdraw a participant from the study has been made solely by the Investigator, who has then had to provide a detailed justification and indicate the line of further management if needed.

In the case of any adverse event, serious adverse event, or adverse drug reaction, the Institutional Ethics Committee (IEC) and Data Safety Monitoring Board are to be informed and participants are to be withdrawn from the study.

### Compliance

During the intervention period, compliance has been evaluated based on the amount of study medication that is consumed. This has been assessed using a compliance assessment form issued to the participants to fill up.

### Concomitant and Rescue Medication

Concomitant therapy has been continued for diabetes mellitus, hypertension, or any other disease, that has not been specifically mentioned in the exclusion criteria**.** The investigators may prescribe any concomitant medications or treatments deemed necessary to provide adequate supportive care during the intervention period**.** The name, indication, dose, unit, frequency, start date, and stop date (if applicable) for all interventions (medicine/procedure/therapy) have been recorded on each participant’s case record form (CRF)**.** Administration of any rescue medicines in case of any medical emergency has been documented in the CRF.

### Outcome Measures

The primary outcome of the study is the change in the DAS-28 score (with ESR) [18] and it is assessed at baseline and the end of the treatment (84th day). Secondary outcomes include the change in disease-specific biochemical and inflammatory markers (RA factor, C-reactive protein, and ESR) and serum immunoglobulin levels (Immunoglobulin G and Immunoglobulin M), participants’ assessment of Pain, Global assessment of disease activity, change in Disability Index score and frequency of use of conventional analgesic/NSAIDs medicines and occurrence of adverse events if any. The change in disease-specific biochemical and inflammatory markers and serum immunoglobulin levels are assessed at baseline and on the 84th day. The participants’ assessments for pain and global assessment of disease activity are assessed using the visual analog scale [19] ranging from 0 mm (no pain) to 100 mm (worst possible pain) at baseline, 28th day, 56th day, and 84th day. The change in Disability Index score is assessed using the Indian Health Assessment Questionnaire [20] at baseline, 28th day, 56th day, and 84th day. The frequency of use of conventional analgesic/NSAIDs medicines and occurrence of adverse events are also assessed in a time frame of baseline, 28th day, 56th day, and 84th day (Figure 1).

### Sample Size

Due to the low prevalence of RA (around 0.7% to 1%) [21], it is not feasible to recruit the participants based on the scientific line of sample size theory. Therefore, based on feasibility and the availability of resources, a total of 240 participants have been recruited from 6 institutes (40 participants at each center), within the given time frame.

### Recruitment

The eligible participants have been screened for inclusion and exclusion criteria from the outreach out-patient departments/ camps/door-to-door visits conducted in the identified area/ village under the selected study sites. The participants have been informed about the study in detail (in their regional language) through banners and IEC materials.

### Allocation

Since the study is conducted in a black box study design and the intervention is classified based on major disease patterns, allocation does not apply to this study.

### Data Collection Methods

Demographic data, clinical history, details of concomitant medications, the score of 2010 American College of Rheumatology and the European League Against Rheumatism criteria, DAS-28, and other assessment parameters of the participants have been recorded in the CRF. The participants were followed up on the 28th day, the 56th day, and the 84th day**.** During follow-ups, the occurrence of any symptom and the need for any rescue medication have been recorded. The contact number of the investigator along with the address of the CCRAS institute have been provided to the participants at the time of enrollment**.** Participants have been instructed to inform about any adverse event that happens during the study period**.**

Besides a hard copy of CRF, the data have been recorded in e-CRF also. All source documentation supporting entries into the CRFs are maintained and will be kept readily available. To ensure the quality, the data are checked for consistency, omissions, and any apparent discrepancies. The reason for withdrawal or dropping out of participation is recorded in the CRF.

### Data Monitoring

A Data Safety Monitoring Board has been constituted to monitor the clinical study and to ensure the safety of the participants. The inspection of various records of the clinical study (CRFs and other pertinent data) will be done by the CCRAS representatives and regulatory authority to ensure strict adherence to the study protocol and correct documentation of the data. Any problems faced by the research staff at the participating site have been addressed timely. The Clinical Monitoring Committee is responsible for verifying the CRFs at regular intervals throughout the study to verify adherence to the protocol; completeness, accuracy, and consistency of the data; and adherence to local regulations on the conduct of clinical research.

### Deviation From the Protocol

Any deviation from the study protocol is implemented in the study only after approval from the IEC.

### Ethical Considerations

The study is conducted according to the national ethical guidelines for biomedical and health research involving human participants (2017) by the Indian Council of Medical Research; and Good Clinical Practice guidelines for clinical trials in Ayurveda, Siddha, and Unani Medicine, 2013. Approval from the IEC of all the concerned institutes has been obtained, and the study has been registered with the Clinical Trial Registry-India (CTRI/2023/06/054203 dated June 20, 2023). The participants have been educated by the investigator verbally and using a written patient information sheet in their regional language and have been asked to provide consent in writing regarding their participation in the study. The participants have been made aware that they are free to leave the study if they wish to discontinue without giving a reason, and without my medical care or legal rights being affected. All the information and records of the study participants including their names and identities are kept confidential in password-protected Microsoft Excel sheets. All the study participants are covered by a clinical trial insurance policy which included coverage for any adverse events occurring during the study.

### Statistical Analysis

After data collection, verification of its accuracy and limits will be done. The filtered data will be used for additional analysis and interpretation. The Categorical variables will be reported in number (percentage) and analysis of pre-post-trial outcomes will be done by the Mc-Nemar chi-square test. The continuous variables (like scores and lab parameters) will be analyzed by paired *t* test/Wilcoxon test as per the distribution of data in the pre-post trial situation. The assessment parameters assigned in more than 2 follow-ups will be analyzed using the r-ANOVA/Friedman test/ Cochran Q test. The continuous data having normal distribution will be represented as mean (SD) and the data not having normal distribution as median (IQR). The 5% level of significance will be used throughout the analysis. The SPSS (version 26.0; IBM Corp) software will be used to conduct the analysis.

## Results

The enrolment of the participants in the study was initiated in December 2023 and the final follow-up was completed in March 2024. Out of 240 participants enrolled, 222 (92.5%) participants have completed the study. Data verification, compilation, and analysis are under process. The findings from the study will be published in peer-reviewed journals after the completion of data analysis.

## Discussion

### Expected Findings

The selected composite Ayurvedic regimen is anticipated to reduce DASs in study participants with RA. This study was designed using a black box approach, which allows for the selection of medicine according to the pattern of disease presentation and associated symptoms instead of evaluating a specific medicine for a particular diagnosis [10].

Ayurvedic approach to treating a disease depends on the breaking of pathogenesis and alleviation of symptoms. The selection of medicines depends on different stages of pathogenesis that are usually assessed by the presentation of the disease. The available studies for the evaluation of Ayurvedic interventions in rheumatoid mostly used study designs such as single-arm studies, non-RCTs, and RCTs using controls from either Ayurveda or modern conventional drugs [22-25]. In these studies, the intervention was the same for all participants diagnosed with RA and did not consider differences in clinical conditions or the relationships with *Dosha* or *Ama*. Furst et al [26] have used modified RCT as a double-blind, randomized, double-dummy design to evaluate the efficacy of Ayurvedic medicines, both alone and in combination with the standard drug, methotrexate. In this study, 148 distinct Ayurvedic formulations with various dosage forms were used, allowing physicians to prescribe the appropriate combination of drugs based on the specific needs of the patient [26]. In our study, we have designed a single-arm black box design that allows the management of RA in different clinical manifestations on the basis of pathogenesis in terms of the association of *Ama* and *Dosha* along with the chronicity of the disease. For this, a predefined composite Ayurvedic regimen was used that is anticipated to reduce DASs in study participants with RA along with a change in disease-specific biochemical markers. Ayush-SG and *Rasnasaptak Kashaya* were given to all participants, while other medications were customized as per the form of disease presentation.

Ayush-SG contains 3 main ingredients as follows: *Shunthi* (*Zingiber officinale* Roscoe), *Guggulu* (*Commiphora wightii* Bhan), and *Godanti Bhasma* (Incinerated calcium sulfate/ gypsum) [12]. *Shunthi* is known to have anti-inflammatory, analgesic, immunomodulatory, and antioxidant activities [27]. Studies show that guggulipid, the oleo-gum resin of the plant *Commiphora wightii*, demonstrates significant antiarthritic and anti-inflammatory effects by targeting key molecular pathways involved in inflammatory responses [28,29]. *Rasnasaptak Kashaya* contains *Shunthi* along with other herbal ingredients such as *Rasna* (*Pluchea lanceolata* Oliver and Hiem), *Gokshura* (*Tribulus terrestris* L), *Guduci* (*Tinospora cordifolia* [Willd] Miers), *Punarnava* (*Boerhaavia diffusa* Linn), *Eranda* (*Ricinus communis* Linn), *Devadaru* (*Cedrus deodara* [Roxb] Loud), and *Aragvadha* (*Cassia fistula* Linn). All these drugs possess anti-inflammatory activities and *Gokshura*, *Eranda*, and *Devadaru* have analgesic properties also. *Rasna* and *Guduci* have antiarthritic and *Aragvadha* has antirheumatic properties additionally [30-36]. A previous study on *Vatari Guggulu*, *Rasnasaptak Kashaya*, and *Brihat Saindhavadi Taila* has shown a significant change in DAS-28, disability index (Indian Health Assessment Questionnaire), and quality of life assessment (SF-36) scores when administered for 12 weeks [37].

*Guduci Ghanavati* contains an aqueous extract of *Tinospora cordifolia,* which possesses antiarthritic, anti-inflammatory, immunomodulatory, and antipyretic properties*.* The in vivo studies on aqueous extract of *T cordifolia* showed significant anti-inflammatory effects comparable with indomethacin and the mode of action is opined to be similar to that of a nonsteroidal anti-inflammatory agent [36].

*Brihat Saindhavadi Taila* is described as effective in pacifying pain in Amavata [37]. It is found effective in alleviating pain, swelling, and tenderness when administered locally in combination with other internal medications [14,38]. Most of the ingredients in this formulation possess anti-inflammatory properties [39-44]. *Eranda,* the base oil in the formulation, contains ricinoleic acid that has shown anti-inflammatory properties on peripheral application in an in vivo study in guinea pigs [45].

*Dashanga lepa* consists of 10 ingredients as follows: *Sirisha* (*Albizzia lebbeck* Benth), *Madhuyashti* (*Glycyrrhiza glabra* Linn), *Tagara* (*Valeriana wallichii* DC), *Raktachandanam* (*Pterocarpus santalinus* Linn), *Ela* (*Elettaria cardamomum* [Linn] Maton), *Jatamansi* (*Nardostachys jatamansi* DC), *Haridra* (*Curcuma longa* Linn), *Daruharidra* (*Berberis aristata* DC), *Kushta* (*Saussurea lappa* CB Clarke), and *Hrivera* (*Pavonia odorata* Willd). Most of the ingredients in this formulation have potent anti-inflammatory properties, and *Tagara* and *Jatamansi* have analgesic action too [46-54]. The anti-inflammatory and analgesic activities of *Dashanga Yoga/lepa* have been demonstrated in an in vivo study conducted on Wistar rats [55].

Anemia is one of the common comorbidities found in RA patients [56]. *Punarnavadi Mandura* contains ingredients such as *Amalaki* (*Phylanthus embelica* Linn), *Danti* (*Baliospermum montanum* Muell – Arg), *Pippali* (*Piper longum* Linn), *Punarnava*, *Kushtha* (*Saussurea lappa* C.B. Clarke) and *Daruharidra* (*Berberis aristata* DC) along with a mineral drug *Mandura bhasma* (incinerated iron oxide). An in vivo study conducted on *Punarnavadi Mandura* has shown significant hematinic activity against mercuric chloride-induced anemia in albino rats [57]. A previous clinical study on the drug also showed significant improvement in serum iron and serum ferritin levels with improvement in the symptoms of anemia such as fatigue, pallor, etc [58].

The pharmacological properties of the ingredients in the formulations and previous studies suggest that the selected composite Ayurveda regimen for the study will be effective in the management of RA. The findings from the study will be disseminated through reputed peer-reviewed journals after the completion of the data analysis. This research represents an initial effort using a black box design, which could lay the groundwork for customized interventions rooted in Ayurvedic concepts of pathogenesis and symptomatology.

### Strength and Limitations

This study is planned using a black box design that will help in providing evidence-based data regarding the effect of personalized medications in Ayurveda for the management of RA. However, the study protocol has some limitations also. The study protocol does not include the assessment of inflammatory markers such as anticyclic citrullinated peptide. In this study, 3 distinct categories are considered for the composite Ayurvedic regimen. Future research could be designed to address a wider variety of disease presentations.

### Future Scope

This study protocol could be extended to similar conditions that are presented with a wide range of symptoms that require a more individualized approach. Future RCTs may be planned to compare the Ayurveda treatment regimen with conventional standard treatment protocol.

### Conclusions

This interventional study that incorporates the black box approach may provide a strong framework for managing RA. This design may be a more reliable method for evaluating the effectiveness and tolerability of the composite Ayurveda regimen in RA.

## Abbreviations

CCRAS: Central Council for Research in Ayurvedic Sciences
CRF: case record form
CTRI: Clinical Trial Registry-India
DAS: disease activity score
ESR: erythrocyte sedimentation rate
HbA1c: hemoglobin A1c
IEC: Institutional Ethics Committee
NSAID: nonsteroidal anti-inflammatory drug
RA: rheumatoid arthritis
RCT: randomized controlled trial
